# Friendly-Sharing: Improving the Performance of City Sensoring through Contact-Based Messaging Applications

**DOI:** 10.3390/s16091523

**Published:** 2016-09-18

**Authors:** Jorge Herrera-Tapia, Enrique Hernández-Orallo, Andrés Tomás, Pietro Manzoni, Carlos Tavares Calafate, Juan-Carlos Cano

**Affiliations:** Department of Computing Engineering, Universitat Politècnica de València, Valencia 46022, Spain; jorherta@doctor.upv.es (J.H.-T.); ehernandez@disca.upv.es (E.H.-O.); antodo@upv.es (A.T.); calafate@disca.upv.es (C.T.C.); jucano@disca.upv.es (J.-C.C.)

**Keywords:** smart cities, human sensors, opportunistic networks, enabling communication technology

## Abstract

Regular citizens equipped with smart devices are being increasingly used as “sensors” by Smart Cities applications. Using contacts among users, data in the form of messages is obtained and shared. Contact-based messaging applications are based on establishing a short-range communication directly between mobile devices, and on storing the messages in these devices for subsequent delivery to cloud-based services. An effective way to increase the number of messages that can be shared is to increase the contact duration. We thus introduce the *Friendly-Sharing* diffusion approach, where, during a contact, the users are aware of the time needed to interchange the messages stored in their buffers, and they can thus decide to wait more time in order to increase the message sharing probability. The performance of this approach is anyway closely related to the size of the buffer in the device. We therefore compare various policies either for the message selection at forwarding times and for message dropping when the buffer is full. We evaluate our proposal with a modified version of the Opportunistic Networking Environment (ONE) simulator and using real human mobility traces.

## 1. Introduction

Opportunistic communications [1,2,3,4,5] are increasingly considered a promising Smart City enabling communication technology. They are applicable to situations when, for example, the cellular network infrastructure has become inefficient due to too many requests, or when no communication infrastructure is available at all [6,7] . Due to the intermittent contacts, Vahdat et al. [8] refer to such networks as Partially Connected Networks. Some authors [9] consider opportunistic networks as a subclass of Delay Tolerant Networks (DTNs) [10].

Communications in mobile opportunistic networks take place upon the establishment of ephemeral contacts among mobile nodes using direct communication via Bluetooth or WiFi direct instead of using the internet infrastructure. Based on this concept, new contact-based messaging applications have recently been developed, such as Firechat (Open Garden, San Francisco, CA, USA) or Meshme (Meshme Inc., New Castle, DE, USA). Firechat, for example, a messaging application initially meant for music festivals, became popular in 2014 in Iraq due to the government restrictions on internet use, and after that during the Hong Kong protests.

In these kinds of disruptive wireless networks, where the communication between mobile devices is ephemeral, and links are typically asymmetric and unstable, sending and receiving information depends on mobility and the opportunity of contacting other devices, as long they are willing to collaborate. The duration of the contacts between the nodes is a key factor in the dissemination of messages; if the contact time is too low, there will not be enough time for nodes to retrieve all pending messages. Moreover, the management of the internal device resources, basically the buffer occupancy, is critical to provide an efficient service.

Authors of [11,12,13,14] offer a wide taxonomy of the protocols designed for these types of networks. The most straightforward solution is the Epidemic protocol [8]. This protocol is widely used as a reference technique, and its operations are based on the store, carry, and forward approach combined with message flooding.

In this paper, we analyse an opportunistic network solution based on a novel messaging diffusion approach, called *Friendly-Sharing*. This approach aims to increase the message delivery probability by interacting with the user in order to extend the contact duration. We specifically focus on evaluating the impact on the effectiveness of this approach for: (1) the buffer size; (2) the buffer management strategy; and (3) the contact duration time. Our objective is to determine the best approach for forwarding stored messages and which method is optimal when the buffer gets full.

The Friendly-Sharing approach aims to increase the contact duration by notifying the users about the time needed to interchange the messages when a new contact starts. This way, the users can decide to wait a little longer, in order to receive (and send) more messages. We implemented this approach in our testbed messaging application GRChat (Unisersitat Politécnica de Valéncia, Valencia, Spain) [15].

By varying the contact duration time, buffer size and message TTL (Time To Live), we compared the performance of the basic Epidemic protocol against the Friendly-Sharing solution by using the ONE (Opportunistic Network Environment) simulator (Helsinki University of Technology, Espoo, Finland) [16]. This simulator was designed and built to specifically evaluate DTN protocols and applications, and focuses on the network layer without considering the details of lower layers such as media access control (MAC) or physical. In order to get realistic results, the movement data is based on the set of human geotagged traces experimentally obtained at the NCCU campus (National Chengchi University, Taipei, Taiwan) [17]; the message generation patterns (frequency and size) are based on statistics related to social networking applications [18]. We also characterise the mobility and the structure of this trace. From this study, we could determine some social patterns of the trace that we used in the performance evaluation.

The performance evaluation showed that our proposal clearly improves the message delivery performance by increasing the delivery success ratio and reducing the delivery time, although extending the message transmission time and introducing some extra overhead in terms of buffer utilisation. Regarding the buffer management strategy, the best policy consists in forwarding the smallest messages first, and in dropping the biggest ones to make room for incoming transmissions.

The outline of the paper is the following: an overview of related works addressing opportunistic networks and message diffusion is presented in Section 2. The description of the Friendly-Sharing proposal and its GRChat implementation is provided in Section 3, while experiments and performance evaluation details are presented in Section 4. Finally, in Section 5, we present our conclusions and future work.

## 2. Related Work

Opportunistic ad hoc networks are characterised by typically intermittent contacts of short duration between pairs of mobile devices. The message delivery probability depends on the contact duration, which is determined by the mobility of the users. The only way to increase this contact duration is by promoting interactions between users. However, people can hold back from cooperating with others for lack of trust or simply to save energy.

In order to improve the delivery ratio, some authors like [19,20,21,22] have proposed new collaboration mechanisms between mobile users. Regarding collaboration or cooperative approaches, we can find research works that encourage collaboration through credit systems. More specifically, in [23,24], the authors affirm that, when a cooperative system offers security and reliability, mobile users are more stimulated to cooperate, especially when the network includes several unknown users. In [25], we can find another method to stimulate the collaboration with the simple idea of giving more credits to those who send more information in a cooperative way. They conclude that a credit-rewarding system stabilises the network, increasing data traffic and cooperation. In this context, some nodes can refuse to cooperate because of security reasons. It is also possible that malicious and/or selfish nodes could cause a negative impact on network performance. Many interesting proposals focused on this topic. In [26], a novel approach is described to detect and avoid selfish nodes using an improved watchdog mechanism [27] that detects selfish nodes with greater accuracy and speed than classical watchdog methods. The authors of [28] considered a decentralised environment and heterogeneous wireless networks. They proposed a distributed algorithm approach with an analytical basis that enables the interaction in the opportunist network according to their needs, while *pseudo-encouraging* collaboration through punishment to selfish nodes.

Considering that a cooperative system is a social opportunistic network where the message diffusion level is crucial, in [29,30], the authors examine a utility-based cooperative data dissemination system where the utility of data is defined based on the social relationships between users. They studied and validated the performance of this system through an analytical model, allowing characterisation of the data diffusion process. Furthermore, they analysed the behaviour of the system with respect to key parameters such as the definition of the data utility function, the initial data allocation on nodes, the number of users in the system, and the data popularity. In this context, the authors of [31] used theoretical analysis applied to social networks to classify and study some diffusion schemes based on the *homophily* (social networks phenomenon) by combining node relationships and their interests in the data. They noticed that, in order to improve diffusion performance, a node should first diffuse, when meeting a friend, the most similar data according to their common interests, while, when meeting a stranger, it should first diffuse the most dissimilar data according to their common interests. A recent paper [32] evaluates the impact of human behaviour in the opportunistic forwarding of messages. Experimental results using the ONE simulator show that, as nodes start to move less (i.e., increasing pause time), fewer messages are expected to be delivered. Since nodes start to encounter each other at a less frequent rate, the number of replications is also expected to decrease. Regarding latency, messages are to experience longer waiting times prior to delivery. An analytical model based on Delay Differential Equations is proposed for the authors of [33,34] to evaluate through simulations the diffusion of messages in social groups taking into account the transmission time of messages. The idea of actively involving users with tasks of minimum impact on their regular activities is also employed by crowd-based participatory systems [35,36].

As stated before, mobility is another key factor affecting message diffusion effectiveness. There are several proposals, such as the ones presented in [37,38,39,40,41], which evaluate the message dissemination behaviour of the Epidemic protocol by focusing on the mobility patterns of the nodes. In these works, the authors explain the relationship between factors such as speed, mobility model, node density, and places. In [42], the authors introduce a new forwarding protocol, HURRy (HUman Routines used for Routing), where the routing decision is based on probabilistic routing techniques like PRoPHET (Probabilistic Routing Protocol using History of Encounters and Transitivity), although it incorporates the contact duration of encounters (unlike previous approaches) to estimate the rating probabilities of all possible paths to a certain destination.

Finally, other studies analyse how the management of mobile device hardware influences the message delivery probability. In the case of buffer management, some authors [43,44,45] evaluate the use of the buffer through priority rules in an attempt to deliver messages without performance loss in the information transmission process.

The aforementioned studies provide an outlook of the most recent research works focused on the analysis and performance improvements for the diffusion of messages by improving collaboration among mobile users. These articles also show the importance of cooperative behaviour in opportunistic networking, and the trend towards models and applications that exploit the particularities of delay-tolerant wireless networks. However, these works do not analyse in an integrated way the performance of the message diffusion taking into account the duration of contact times, the buffer management schemes, and the relation between the contact duration time and the message size.

## 3. The Friendly-Sharing Approach

In this section, we detail the implementation of the Friendly-Sharing scheme that is based on the Epidemic Protocol. Then, we detail the buffer management policies considering both the forwarding of the stored messages, and the message dropping when memory for new messages is required.

### 3.1. Message Diffusion

Message diffusion is based on epidemic dissemination, a concept similar to the spreading of infectious diseases. Basically, when an infected node (i.e., a node that has a message) contacts another node, it infects it by transmitting the message. Epidemic dissemination obtains the minimum delivery delay at the expense of increased buffer usage and increased number of transmissions. The critical factor affecting diffusion is the duration of the contact, which depends on the mobility patterns of the user. It can vary from a few seconds (a short contact in the street) to hours (for example, during a class at school), and it determines the number and size of the messages that can be exchanged.

A way to improve the effectiveness of this diffusion is to increase the number and duration of contacts. Our proposal, called Friendly-Sharing, has to be integrated into mobile applications that will therefore ask the user to wait for some more time to extend the exchange window. The basic assumption is that installing and being interested in using such an application indicates that the user wants to actively collaborate in the diffusion of the information. Moreover, our proposal allows the applications to inform the users about how much time is needed for interchanging the messages, thus providing a friendly way to stop for a short time. We demonstrate our proposal with a proof-of-concept application, called GRChat [15].

**Figure 1:**
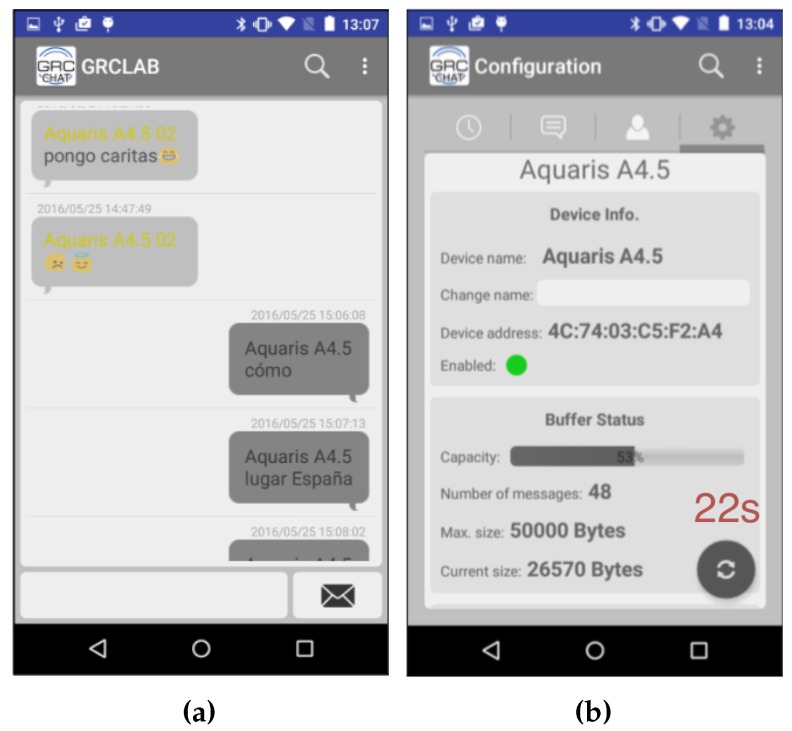
Several screenshots of the GRChat app. (**a**) a typical chat conversation; and (**b**) status of message interchange, showing the remaining time for end the transmission.

GRChat is an Android app that can establish connections among two or more phones and transmit data and images using Bluetooth. Moreover, the user can create and subscribe to groups in order to send/receive messages. GRChat works like any messaging app where the user can watch previous messages/images and write new ones. When the user pushes the send button, GRChat connects to any nearby devices and sends the newly created message, as shown in Figure 1a; meanwhile, it also receives the new messages from the other devices. When a device gets a new message, it also tries to connect to other devices in order to complete the diffusion of the message. When no messages are sent, the application is periodically searching for nearby devices.

When the GRChat app detects a new device, it establishes a new pairwise connection and automatically starts the interchange of messages. The application notifies both users about the new connection playing a sound or vibrating. This way, the users can decide to wait some extra time in order to extend the message sharing session. The required time to complete the message interchange is shown in the application, so both users are aware of the pending time (see Figure 1b). This is an effective way to increase cooperation as the user knows exactly the time it should wait. Nevertheless, due to the cooperative approach, users are not forced to wait until all messages are exchanged.

We evaluate three different user behaviours:

- *No-wait*: The users do not stop any additional time. In this case, the number of messages exchanged between nodes will depend on how long they remain within the communication range and clearly on the data rate of the channel. If this contact duration is very small, no messages will be transmitted.
- *Full-wait*: The users wait during the time required to exchange all the messages. In this case, the owner of each mobile device will control this exchange by stopping and waiting until the message transmission is fully completed.
- *Finite-wait*: To avoid interfering too much with the user mobility, users wait only for some extra time to send and receive some of the pending messages.

### 3.2. Buffer Management

**Figure 2:**
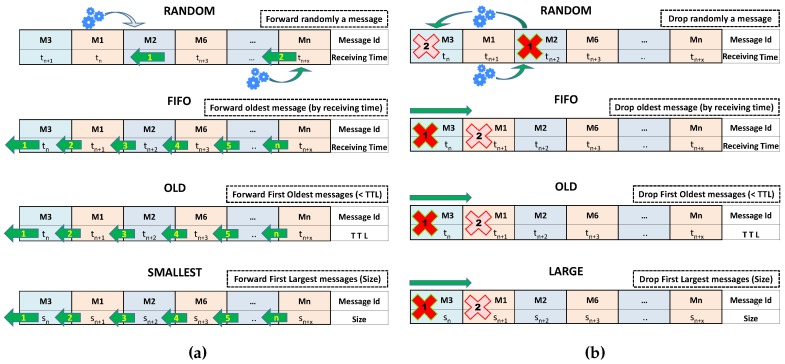
Message forwarding (**a**) and dropping (**b**) policies in the local buffer.

It is important to select the order of the messages sent when two nodes are sharing their messages, especially when users decide not to wait until all messages are sent. Furthermore, we must be aware that both the buffer size and the channel bandwidth are limited. It is, therefore, important to define mechanisms to optimise the use of these resources in a mobile device. In this subsection, in order to evaluate the impact of the forwarding methods on the messages delivery probability and latency, we describe some approaches related to the messages forwarding and messages dropping process (see Figure 2).

#### 3.2.1. Message Forwarding

Message forwarding refers to the order for extracting messages from the buffer in order to transmit them to the connected device. We considered the four methods graphically described in Figure 2a. More specifically:

1. *Random:* The messages are selected randomly from those stored in the buffer. This method is already implemented in the ONE simulator and it is configured by default.
2. *FIFO (First-In, First-Out):* Messages are selected for forwarding according to their arrival order, i.e., the first message to be forwarded is the first message arriving at the mobile device (that is the oldest message, taking into account the reception time). From the figure: message *M3* arriving at time $t_{n}$ will be forwarded first, message *M1* arriving at time $t_{n+1}$ will be the next one to be sent, *M1* will be the following, and so on.
3. *Oldest:* With this approach, the message with lower TTL value will be forwarded first. In the example shown in the Figure, *M3* will be sent first, the next message will be *M1* with TTL value $t_{n+1}$, etc. (note, that this approach is different to the *FIFO* approach, as *FIFO* considers the arrival time of the message, while *Oldest* considers the TTL.)
4. *Smallest:* This approach aims at first sending the smallest messages. In the figure, we can see that the sending queue is organized according to the messages size, i.e., *M3* with size $S_{n}$ will be forwarded first than message *M1* with a message size of $S_{n+1}$, since $S_{n}<S_{n+1}$.

On the original version, ONE only implements the *Random* and *FIFO* policies. The other two policies were implemented by the authors, as detailed in the following section.

#### 3.2.2. Message Dropping

Message dropping refers to the order followed when making room for a new arriving message. For this, we need to determine which message or messages have to be dropped in order to make room for the incoming message. This operation is called only when the buffer is full. Figure 2b shows the approaches we considered. More specifically:

1. *Random:* The message or messages to be removed from the buffer are selected randomly.
2. *FIFO (First-In, First-Out):* The first message to be removed from the buffer is the oldest message arriving at the mobile device, taking into account the reception time. From the figure: message *M3* that arrived at time $t_{n}$ will be removed first, and message *M1* with arrival time $t_{n+1}$ will be the next to be deleted.
3. *Oldest:* With this approach, the message with lower TTL value $t_{n}$ will be removed from the buffer. In the example in the Figure, *M3* will be the first, the next message to be dropped will be *M1* by having TTL value $t_{n+1}$, etc.
4. *Largest:* This approach removes the largest messages. In the figure, we can see that the dropping queue is organised according to the message size, i.e., *M3* with size $S_{n}$ will be deleted first than message *M1* with a message size of $S_{n+1}$.

Since only the FIFO method was implemented in the ONE simulator, we implemented the three other policies.

## 4. Performance Evaluation

In this section, we evaluate the efficiency of our proposed Friendly-Sharing approach. As commented previously, the performance of the message diffusion will mainly depend on the mobility of the users and the number of contacts and their duration between pair of users. The impact of the dropping and forwarding policies in the local buffer is also evaluated, in order to select the best policy.

**Figure 3:**
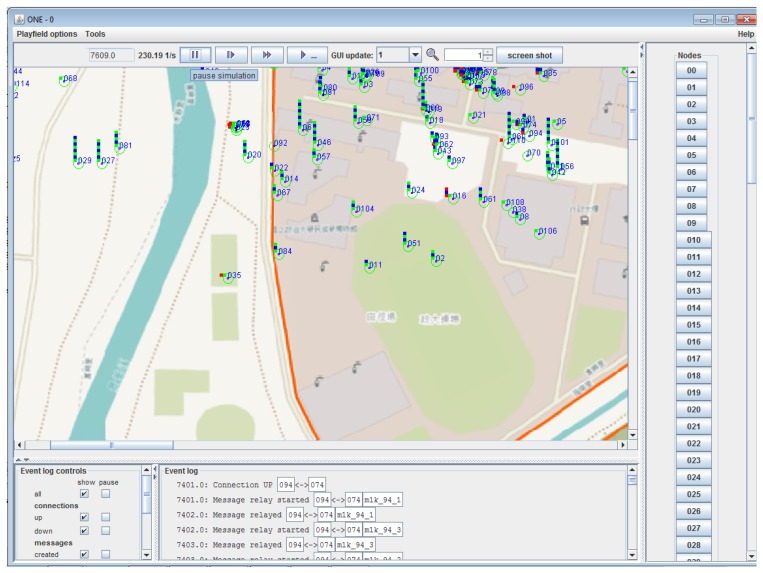
The ONE simulator (Helsinki University of Technology) running with the NCCU (National Chengchi University) traces.

We employed the ONE simulator [16] using a real movement trace of mobile users. This trace comes from an experiment at the NCCU campus [17], where GPS position data was collected during two weeks (336 h) using an Android app installed in the smartphones of 115 students. Figure 3 shows a snapshot of the ONE running with the corresponding graphical map information from NCCU.

The workload considered tried to mimic the typical data-flow for a multimedia messaging application where shorter messages are far more common than larger ones. Three message sizes and frequencies were considered: (1) a short text message (1 kB) every hour; (2) a photo (1 MB) every 18 h; and (3) a video or high-resolution picture (10 MB) every 96 h. These frequencies were based on the statistics of Whatsapp (Facebook, Inc., Menlo Park, CA, USA) message usage from [18], while sizes are approximations of the content produced by current mobile phone hardware.

The communication range (*r*) was set to 7.5 m with bandwidth $B_{w}=2.1$ Mb/s. These assumptions allow taking into consideration the necessary set-up time of connections and the possible impact of interferences. These values are based on the Bluetooth 2.0 class 2 specifications. Other technologies such as WiFi Direct are not considered to avoid issues like disconnecting the device from the internet. WiFi is increasingly being used for data connectivity, especially at large enterprise areas or university campuses, and it would be a nuisance for users to deal with these disconnection periods.

### 4.1. ONE Simulator Modifications

The ONE simulator was designed and built specifically to assess protocols for message dissemination in DTN Networks, namely: Epidemic, Spray and Wait, Prophet, First Contact, Direct Delivery, and Maxprop. ONE can use real traces or synthetic mobility models like Random Walk, Random Way Point, Grid and Linear. These mobility models can be combined to model complex behaviours with different patterns as the day progresses (like office and work days).

The ONE buffer management only includes FIFO and random policies, so we extended it with the other two criteria considered in this paper: TTL and message size. The Friendly-Sharing approach was implemented by stopping the nodes that start a transmission. This movement pause lasts for a maximum time specified in the simulation parameters. After this pause, both nodes continue their original trajectories following the trace movement. The original ONE message generation interval follows a uniform random distribution. In order to obtain a more realistic model, we implemented an independent Poisson process for each user and message type.

**Figure 4:**
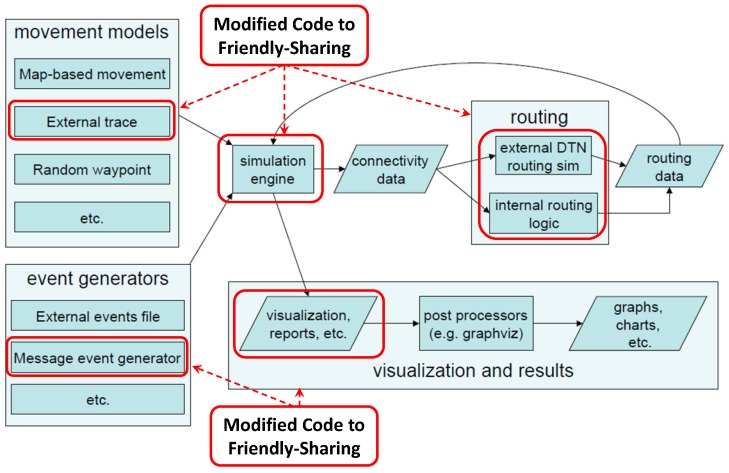
Modifications to the ONE simulator code (original figure from [16]).

The required modifications are summarised in Figure 4. In order to implement the Forced Stop approach and the buffer management policies, we modified the ActiveRouter, DTNHost, MessageRouter and Connection Java classes. We added the ExponentialMessageGenerator to inject messages using an exponential distributed random interval. We also implemented the function drop-to-insert in order to implement the dropping police when the buffer is full. Finally, we added two subclasses of Report to obtain the buffer occupancy and transmission time information.

### 4.2. Trace Characterisation

In order to characterise mobility and determine the structure of the contacts, we analysed the trace used in the experiments. From this study, we could determine some social patterns of the trace that are useful to understand the performance of the Friendly-Sharing approach.

**Figure 5:**
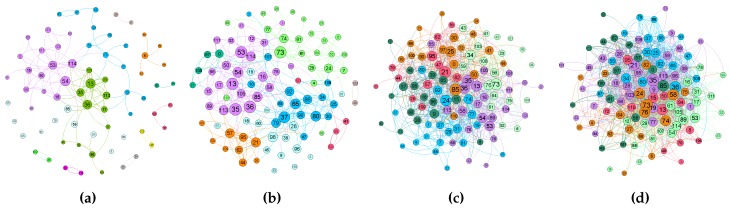
Contact graph for different time intervals of the trace: (**a**) 3 h; (**b**) 6 h; (**c**) 12 h and (**d**) 24 h.

Figure 5 shows the contact graph for different time intervals, starting from the first 3 h of the trace up to 24 h. Each node is labelled by a number and the diameter of each circle is proportional to the number of contacts. Node groups are represented by different colours and were computed using the Fruchterman–Reingold algorithm [46]. We can see that the social interrelation given by the dynamics of the contacts numbers increases according to the simulation time, while the number of communities decreases: on the first 3 h of simulations, the number of communities are 12, for 6 h and 12 h, we have eight communities, and, finally, after 24 h, we only have six communities. This means that the number of “communities” slowly decreases, thus contributing to message diffusion and all nodes eventually getting connected. This implies increasing the message TTL to 12 h or 24 h in order to produce a better delivery rate.

**Figure 6:**
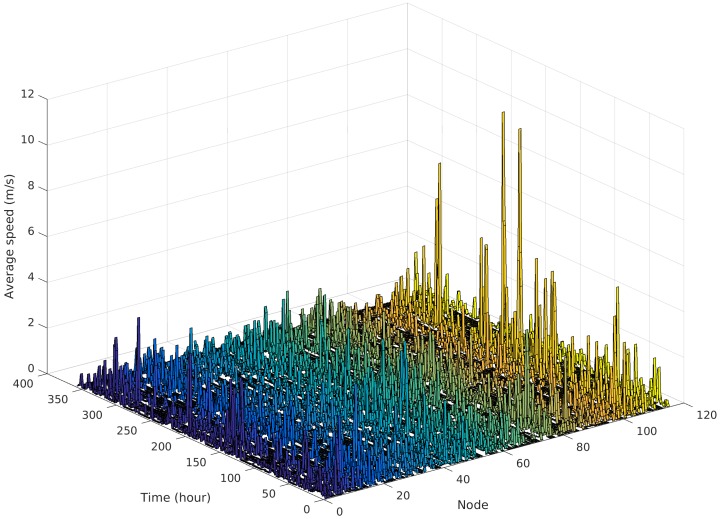
Average node speed at each hour of the movement trace.

Figure 6 presents the average node speed at each hour of the movement trace, clearly showing that nodes have very dissimilar movement patterns. Some nodes move frequently and with considerable speed, while other nodes are quite static and move with lower speed. This effect could be explained by the students place of residence, e.g., some sleep close to the campus (or even inside) while others have to travel further away to their homes.

**Figure 7:**
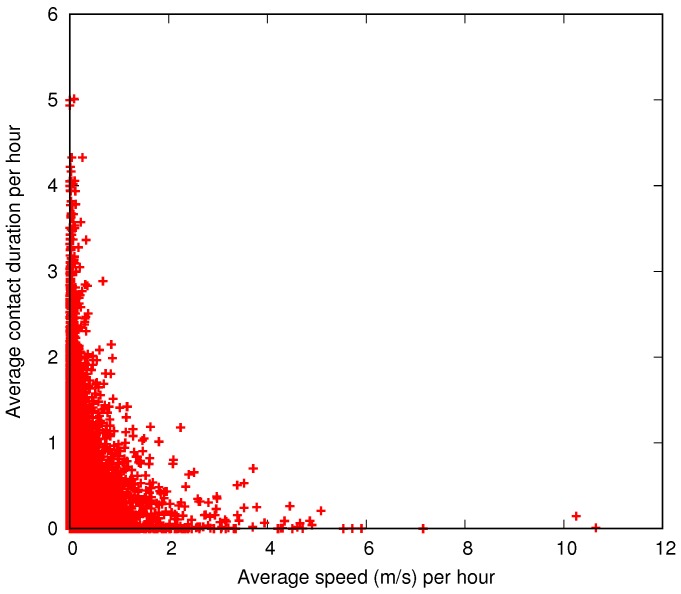
Average speed and number of contacts for all nodes at each hour of the movement trace.

Although nodes without movement in a large period of time could be considered not relevant for message interchanges, Figure 7 shows exactly the opposite. In this plot, the *x*-axis shows the average speed computed as in Figure 6, while the *y*-axis shows how much time a node is in contact during an hour. For example, a node that has an average speed of 2 m/s and a total contact duration of 1 h across all contacts will be plotted in position (2,1). As a node could be contacted by more than one node at the same time, the *y*-axis extends away from 1. This inverse correlation between movements and contacts is easily explained by the students’ typical behaviour: they stay together in a class for a bounded amount of time, they move a small distance to the next class, and so on. Even while not attending classes, they spend time with other students in the cafeteria, library, or other campus services.

### 4.3. Buffer Management Evaluation

In this subsection, we evaluate the different buffer management policies for different buffer sizes, in order to select the best combination. Several simulations were executed for all the 16 possible combinations using the epidemic protocol. Each forwarding and dropping combination was tested with a message TTL of 12 or 24 h, and buffer sizes of 50 MB, 100 MB, 200 MB and 1 GB. These simulation parameters are summarised in Table 1.

**Figure 8:**
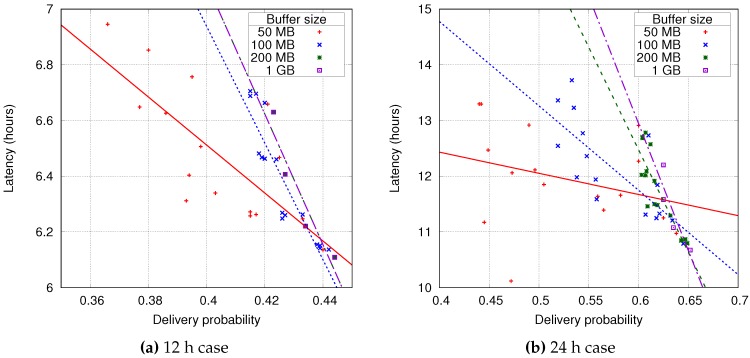
Delivery probability versus latency for a 12 and 24 h TTL (Time To Live) with different buffer sizes and queue policies.

Figure 8a,b show the relation between delivery probability and average message latency for each of the simulations. This plot uses a point for each buffer management policy combination with the same symbol for a particular buffer size. It shows that there is a clear correlation between delivery probability and latency and that, as the buffer size increases, the variability of the delivery probability decreases. This last fact is even more evident when increasing the TTL for the messages, where a minimum of a 200 MB buffer is required to stabilise this metric.

The main factor for a good delivery probability is how long messages are available in the network (TTL), for larger times, the probability of arrival increases. The buffer size also plays an important role, as the buffer size increases the buffer management policies have a smaller impact on the message delivery. When the buffer size is large enough to keep almost all messages, the dropping policy is no longer relevant. Table 2 and Table 3 show the delivery probability for each policy combination, where the best values are for the combination of *smallest* forwarding and *largest* dropping policies. In this case, the buffer management policies are giving priority to the small messages, which also have a better transmission chance in a short contact time.

### 4.4. Friendly-Sharing Diffusion Evaluation

In this section, we study the benefits of the proposed Friendly-Sharing diffusion approach compared to the standard epidemic protocol. For the sake of brevity, we will focus on the smallest/largest buffer management policies as the previous subsection demonstrates that they obtain the best delivery probability. We repeat the same simulation parameters schema with different buffer sizes and TTL times, summarised in Table 4.

**Figure 9:**
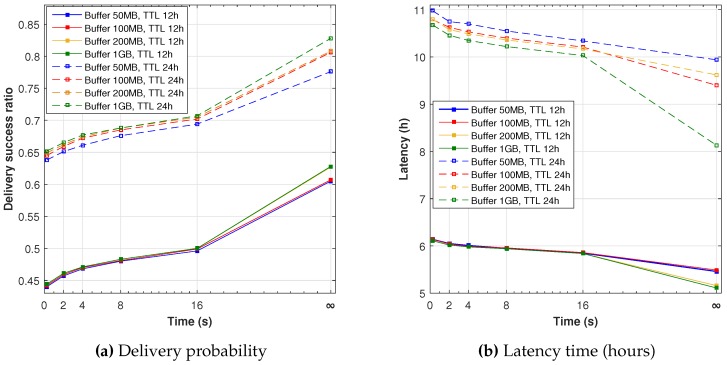
Average delivery success ratio and latency.

Figure 9a shows the delivery ratio of opportunistic networks in relation to the Friendly-Sharing maximum time. A value of 0 in the *x*-axis represents the epidemic protocol without any additional wait, and a value of ∞ refers to the *full-wait* approach, where nodes wait until they transmit all messages in their buffers. The experiment shows, as expected, that waiting until all messages are interchanged improves the message diffusion. In this case, the epidemic protocol has a delivery probability less than 0.45, while increasing the wait time from 2 s to 16 s the delivery probability improves to 0.50. The best probability appears for the *full-wait*. As in the previous experiments, the TTL parameter is the main delivery performance factor, showing that an increase from 12 to 24 h improves the delivery probability by about 20%. Using a bigger buffer has little impact with a TTL of 12 h and no discernible improvement when the TTL is configured to 24 h.

Regarding latency, comparing both plots in Figure 9b, it is clear how it decreases as the delivery probability increases for each TTL value. This validates our previous assumption of an approximate inverse relation between the two measures. Following this relation, the buffer size does not have much impact on latency, and the TTL continues to be the main factor.

These experiments show a clear trade-off between delivery probability and latency. Allowing a large TTL greatly improves the delivery probability, it also increases the latency. Roughly, doubling the TTL from 12 to 24 h improves the delivery probability by 50%, but the latency is also doubled. This limit on the routing performance is due to the movement traces themselves, which dictates how much time it takes to contact, from one node to another.

**Figure 10:**
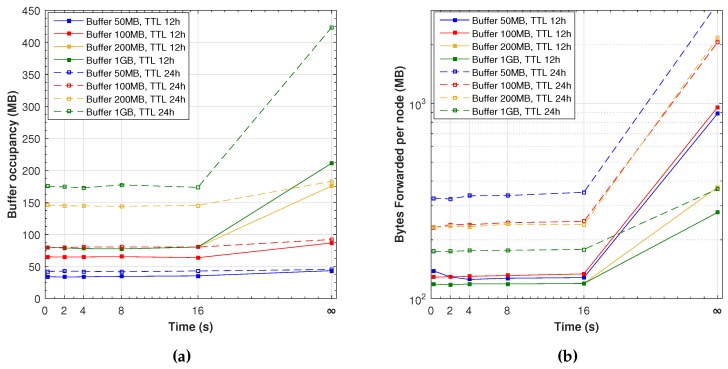
Overhead results: Buffer occupancy and forwarded bytes. (**a**) maximum of the average buffer occupancy from each node; and (**b**) average bytes daily forwarded per node (*y*-axis in log scale).

Considering protocol overhead, Figure 10a shows the maximum of the average buffer occupancy from each node. With the simulated workload, a buffer of around 80 and 175 MB seems sufficient for a TTL of 12 and 24 h, respectively. Bigger buffers are only required when simulating the ideal scenario with indefinite pauses. It is interesting to note that even small additional waiting times improve the delivery probability and latency without increasing the buffer overhead.

In epidemic protocols, another overhead measure is the amount of information forwarded per node. Figure 10b shows the daily average bytes forwarded by each node, which is the sum of all bytes transmitted divided by the number of nodes and the simulation length in days. The number of transmissions increases greatly with a large TTL time, basically because more messages stay in the buffers during more time. A big buffer avoids unnecessary retransmissions; therefore, it also reduces the time required by nodes to stop waiting to complete the data transfer. This protocol does not provide any reception acknowledgements; therefore, nodes keep forwarding messages that have already reached their destination, up to the maximum of TTL time.

Similarly to the previous buffer occupancy analysis, it is interesting to point out that even making small movement pauses with our Friendly-Sharing improves the delivery probability and latency without much transmission overhead.

**Figure 11:**
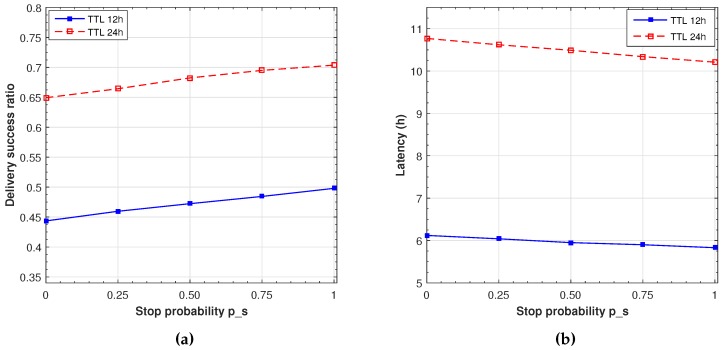
Delivery ratio (**a**) and latency (**b**) for different contact stop probabilities (16 s max. stop).

In our previous analysis, a full cooperation is assumed, since, as indicated before, we suppose that installing the application shows that the users are willing to follow its indications. As reasonable as this argument could be, there are cases where users may not want to stop or simply cannot stop. Furthermore, users will stop longer when contacting other related users or depending on past interactions; that is, stopping probability and duration has a strong social dependence. However, no social data can be easily extracted from the movement traces. We reflect this situation through the *probability of stopping* ($p_{s}$). This probability ranges from 1, which reflects full cooperation of both users (as considered in the previous evaluations) to 0, indicating no cooperation (and no waiting time, which is the basic epidemic protocol). In order to evaluate the impact of this probability, we set the buffer size to 200 MB considering 16 s maximum stop time for both TTL (which is one of the experiments represented in Figure 9). We evaluated the delivery success ratio and latency for different values of the stop probability. This probability is modelled as a uniform distributed random variable independent for each contact. The results are presented in Figure 11, showing a linear correlation between stop probability and delivery success and latency. Moreover, we can see that stopping 16 s with a stopping probability of 0.5, we obtain a similar delivery and latency performance as with a 8 s unconditional stop, as displayed in Figure 9. A similar result can be observed for a probability of 0.25, which corresponds to 4 s, and for a probability of 0.75, that corresponds to 16 s. We can, therefore, conclude that the effect of the stop probability is equivalent to reducing the waiting time by a factor $p_{s}$.

## 5. Conclusions

Contact-based communications are increasingly considered a promising Smart City enabling communication technology. In this paper, we introduced a new diffusion approach, Friendly-Sharing, where users are informed about the remaining time needed to interchange the messages stored in their buffers. In this way, the user can decide to wait longer in order to increase the number of messages exchanged, thus increasing the delivery rate.

We used the ONE simulator with real human mobility traces and realistic message generation patterns based on social networking applications to evaluate the performance of the Friendly-Sharing diffusion. The behaviour of students produced interesting contact patterns: nodes with low mobility have more contacts than high mobility ones.

We showed that the greater the number of messages in local buffers, the better the diffusion (in terms of delivery probability and latency). Thus, a key aspect for efficiency is the buffer management policy, especially when there is no full message interchange. The experiments show that the best results were obtained when forwarding the smallest messages first and dropping the biggest ones to make room for incoming transmissions. Regarding the waiting time for Friendly-Sharing, we also determined that when nodes wait even an extra two seconds each time they contact, it improves the delivery performance while not having a relevant impact on the buffer occupancy or on the protocol overhead.

As a proof-of-concept, we showed that Friendly-Sharing can be easily introduced in messaging applications and can effectively improve the collaboration, increasing the delivery ratio and reducing the latency of message delivery with a limited impact on buffer utilisation.

## Figures and Tables

**Table 1:** Simulation parameters varied to evaluate queue management policies.

Parameter	Values
Buffer Size	50 MB, 100 MB, 200 MB, 1 GB
Time to Live	12 h, 24 h
Forward policy	Random, FIFO, Oldest, Smallest
Discard policy	Random, FIFO, Oldest, Largest

**Table 2:** Delivery probability for a 12 h TTL (Time to Live) with different buffer sizes and queue policies.

Buffer Size	Drop Policy	Forwarding Policy			
		Random	FIFO	TTL	Smallest
50 MB	Random	0.394	0.386	0.380	0.415
	FIFO	0.403	0.398	0.395	0.415
	TTL	0.393	0.377	0.366	0.417
	Largest	0.433	0.425	0.421	0.440
100 MB	Random	0.426	0.419	0.415	0.439
	FIFO	0.427	0.420	0.417	0.438
	TTL	0.426	0.418	0.415	0.439
	Largest	0.433	0.424	0.420	0.442
200 MB	Any	0.434	0.427	0.423	0.444
1 GB	Any	0.434	0.427	0.423	0.444

**Table 3:** Delivery probability for a 24 h TTL (Time to Live) with different buffer sizes and queue policies.

Buffer Size	Drop Policy	Forwarding Policy			
		Random	FIFO	TTL	Smallest
50 MB	Random	0.473	0.449	0.442	0.559
	FIFO	0.505	0.496	0.490	0.565
	TTL	0.472	0.445	0.440	0.582
	Largest	0.625	0.600	0.600	0.638
100 MB	Random	0.538	0.519	0.519	0.607
	FIFO	0.557	0.548	0.535	0.618
	TTL	0.558	0.544	0.533	0.621
	Largest	0.634	0.619	0.610	0.645
200 MB	Random	0.609	0.603	0.604	0.643
	FIFO	0.616	0.607	0.604	0.643
	TTL	0.619	0.608	0.607	0.647
	Largest	0.632	0.616	0.612	0.649
1 GB	Any	0.635	0.625	0.625	0.652

**Table 4:** Simulation parameters varied to evaluate diffusion approaches.

Parameter	Values
Buffer Size	50 MB, 100 MB, 200 MB, 1 GB
Routing	Epidemic, Friendly-Sharing
Maximum wait time	2 s, 4 s, 8 s, 16 s, ∞
Time to Live	12 h, 24 h
